# Pathological Features of Enterovirus 71-Associated Brain and Lung Damage in Mice Based on Quantitative Proteomic Analysis

**DOI:** 10.3389/fmicb.2021.663019

**Published:** 2021-06-18

**Authors:** Yuefei Jin, Dong Li, Tiantian Sun, Yue Du, Yanlei Gao, Ronghua Ding, Wangquan Ji, Weiguo Zhang, Haiyan Yang, Shuaiyin Chen, Guangcai Duan

**Affiliations:** ^1^Department of Epidemiology, College of Public Health, Zhengzhou University, Zhengzhou, China; ^2^Department of Immunology, Duke University Medical Center, Durham, NC, United States; ^3^Henan Key Laboratory of Molecular Medicine, Zhengzhou University, Zhengzhou, China

**Keywords:** enterovirus 71, quantitative proteomics, central nervous system, pulmonary edema, complement and coagulation cascades

## Abstract

The outbreaks of enterovirus 71 (EV71)-associated hand, foot, and mouth disease (HFMD) have emerged as an emergency of global health due to its association with fatal encephalitis and subsequent neurogenic pulmonary edema; however, the molecular characteristics and pathological features underlying EV71-associated encephalitis and pulmonary edema remain largely unknown. In this study, we performed a proteomic analysis of fresh brain and lung tissues from EV71-infected mice at 7 days post infection. We detected a perturbed expression of 148 proteins in the brain and 78 proteins in the lung after EV71 expression. Further analysis showed that the dysregulated proteins in the brain are involved in a variety of fundamental biological pathways, including complement and coagulation cascades, innate and adaptive immune responses, platelet activation, and nitrogen metabolism, and those proteins in the lung participate in innate and adaptive immune responses, phagosome, arginine biosynthesis, and hypoxia-inducible factor 1 signaling pathway. Our results suggested that immune activation, complement and coagulation dysfunction, platelet activation, imbalance of nitrogen metabolism, and hypoxia could be involved in the pathogenesis of EV71, which explains the major clinical manifestation of hyperinflammatory status of severe HFMD cases. Our study provides further understanding of the molecular basis of EV71 pathogenesis.

## Introduction

Hand, foot, and mouth disease (HFMD) is a common childhood illness caused by human enteroviruses belonging to the *Enterovirus* genus in the *Picornaviridae* family, characterized by blister-like sores or rashes on the hands, feet, and mouth (Solomon et al., 2010). Among these enteroviruses, enterovirus 71 (EV71) is one of the major pathogenic agents of HFMD (Solomon et al., 2010). In 1969, EV71 was first isolated from the stool specimen of pediatric patients with central nervous system (CNS) disease in California (Schmidt et al., 1974). Since then, outbreaks or epidemics of EV71 have been frequently reported in a range of countries worldwide (Solomon et al., 2010). The outbreaks in recent years mainly occurred in the Asia-Pacific region, including China (Ho et al., 1999; Yang et al., 2017), Singapore (Ang et al., 2015), Malaysia (Chan et al., 2000), Vietnam (Van Tu et al., 2007), and Japan (Mizuta et al., 2005). Sporadic cases were also frequently reported from most European countries, such as France (Mirand et al., 2016), United Kingdom (Teo et al., 2019), Germany (Karrasch et al., 2016), and Italy (Neri et al., 2016), suggesting that EV71 becomes a global health threat. Most EV71 infections are generally mild and do not require special antiviral treatment, but some cases can develop into severe complications, such as aseptic meningitis, acute flaccid paralysis, and neurogenic pulmonary edema (Solomon et al., 2010). In severe cases, the disease is characterized by sudden deterioration, rapid progression, high mortality, and neurological sequelae (Solomon et al., 2010). Between 2008 and 2012, 2,457 fatal cases were reported in China alone (Xing et al., 2014). In 1997, 2000, and 2006, 41, eight, and six deaths caused by EV71 were reported in Malaysia, respectively (Chua and Kasri, 2011). From 1998 to 2010, about 250 patients died of EV71 infection in Taiwan, China (Wang et al., 2012a). As a matter of fact, EV71 has been considered as the most severe neurotropic virus in the post-polio era. Although EV71-inactivated vaccine has been approved by the China Food and Drug Administration (Mao et al., 2016), the incidence of HFMD remains high. To date, no effective antiviral treatment is available to treat severe infections except symptomatic therapies. Therefore, there is still a long way to go before reaching effective protection against HFMD severity.

Established evidence suggests that EV71 can invade the CNS system through a disrupted blood–brain barrier (BBB) or retrograde axonal spread, leading to CNS lesion (Solomon et al., 2010). During the EV71 epidemic in the Asia-Pacific region, a large number of infections with severe neurological disease were recorded (Ho et al., 1999; Mizuta et al., 2005; Van Tu et al., 2007; Chua and Kasri, 2011; Xing et al., 2014). Children younger than 5 years old possess the highest incidence of CNS disease (Chang et al., 2002). Evidence from imaging and histopathological analysis indicates that the neurological complications and neural disorders in EV71 infections may be associated with inflammation in the CNS region, including the cerebral cortex, the brain stem, and the spinal cord (Lum et al., 1998; Huang et al., 1999; Chen et al., 2001; Huang, 2001). The pathological features of CNS damage caused by EV71 are described as perivascular cuffs, variable edema, neuronophagia, and microglia nodules, which are extremely similar to those in encephalitis induced by other viruses (Solomon et al., 2010). Pulmonary edema and hemorrhage were other severe complications observed in EV71 infections, resulting in the highest mortality (Chang et al., 1999, 2002; Chan et al., 2000; Chen et al., 2001). Several autopsy reports on EV71 patients who died from pulmonary edema showed that inflammation was only observed in the CNS region, not detected in the lung and heart (Lum et al., 1998; Shieh et al., 2001; Chang et al., 2007). These lines of evidence suggest that EV71-induced pulmonary edema may be a neurogenic origin, a secondary effect of autonomic dysfunction due to infection within the brainstem. Although this acute lung injury was preceded by and closely related to the CNS damage, our knowledge on EV71 pathogenesis in the brain remains largely unknown.

Biomacromolecules (typically polypeptides/proteins) are the key participants in many cellular processes. Their composition, trafficking, and interactions underlie the dynamic processes of human life (Li et al., 2017). Diseases are often accompanied by the malfunction of proteins at multiple levels. Exploring how biological procedures are regulated at the protein level is of great importance to understand the molecular basis for viral pathogenesis (Aslam et al., 2017; Li et al., 2017). In the present study, we used mass spectrometry (MS)-based proteomics to identify proteins whose expression was affected by EV71 infection in mice. Our study will provide further understanding of EV71 pathogenesis.

## Materials and Methods

### Ethics Statement

The experimental animals were inbred, specific-pathogen-free BALB/c mice. All animal experiments were approved by the Life Science Ethics Review Board of Zhengzhou University (permission no. ZZUIRB2020-29).

### Mice

The BALB/c mice used in this study were obtained from the Experimental Animal Center of Zhengzhou University, and all mice were housed in a specific-pathogen-free facility of the College of Public Health of Zhengzhou University on a 12-h light/dark cycle, with *ad libitum* access to food and water.

### Animal Infection Experiments

As described in our previous study (Jin et al., 2018a), 3-day-old BALB/c mice were inoculated intraperitoneally (i.p.) with 2 × 10^6^ pfu EV71 (ZZ1350 strain, KY886010.1). The control mice were inoculated with an equal volume of the culture supernatant of human rhabdomyosarcoma (RD) cells and kept in a separate cage. Brains and lungs were removed from euthanized mice at 7 days post-infection (dpi) for quantitative proteomics (*n* = 4 for control-infected mice; *n* = 4 for EV71-infected mice), immunohistochemical (IHC) (*n* = 2 for control-infected mice; *n* = 2 for EV71-infected mice), and Western blotting analyses (*n* = 2 for control-infected mice; *n* = 2 for EV71-infected mice). The IHC and Western blotting data shown are representative of three separate experiments.

### Tissue Protein Isolation

The tissue samples were fully homogenized with an electric tissue homogenizer on ice. After that, RIPA lysis buffer (1 ml/100 mg) containing 1% protease inhibitor cocktail was added to the samples. After lysis on ice for 1.5 h, the debris was removed by centrifugation at 12,000 *g* at 4°C for 10 min. Finally, the supernatant was collected, and the protein concentration was determined with a BCA kit (Thermo) according to the manufacturer’s instructions.

### Enzymatic Digestion

For digestion, the protein extracts were reduced with 5 mM dithiothreitol (Sigma Aldrich, St. Louis, MO, United States) for 30 min at 56°C and alkylated with 11 mM iodoacetamide (Sigma Aldrich) for 15 min at room temperature in darkness. The urea concentration of the sample is diluted to less than 2 M. Finally, trypsin was added at 1:50 trypsin-to-protein mass ratio for the first digestion overnight and 1:100 trypsin-to-protein mass ratio for a second 4-h digestion.

### TMT Labeling

After trypsin digestion, the peptides were desalted by Strata X C18 SPE column (Phenomenex) and vacuum-dried. The peptides were then reconstituted in 0.5 M TEAB and processed according to the manufacturer’s protocol for TMT kit. Briefly, one unit of TMT reagent was thawed and reconstituted in acetonitrile. The control-infected samples were labeled with 126, 127N, 127C, and 128N and the EV71-infected samples were labeled with 128C, 129N, 129C, and 130N. The peptide mixtures were then incubated for 2 h at room temperature and pooled, desalted, and dried by vacuum centrifugation.

### HPLC Fractionation

The TMT-labeled peptides were fractionated by high-pH reverse-phase HPLC using Agilent 300Extend C18 column (5 μm particles, 4.6 mm ID, 250 mm length; Supelco, Bellefonte, PA, United States). Briefly, the peptides were first fractionated with a gradient of 8 to 32% acetonitrile (pH 9.0) over 60 min into 60 fractions. Then, the peptides were noncontiguously pooled into 18 fractions and dried by vacuum centrifugation.

### LC–MS/MS Analysis

The dried samples were dissolved in 0.1% formic acid (solvent A) and directly loaded onto a homemade reversed-phase analytical column (15 cm length, 75 μm ID; Thermo). The gradient was comprised of an increase from 6 to 23% solvent B (0.1% formic acid in 98% acetonitrile) over 26 min, 23 to 35% in 8 min, climbing to 80% in 3 min, and then holding at 80% for the last 3 min, all at a constant flow rate of 400 nl/min on an EASY-nLC 1000 UPLC system (Thermo).

The peptides were subjected to nanospray ion source followed by tandem mass spectrometry (MS/MS) in Q ExactiveTM Plus (Thermo) coupled online to the UPLC. The electrospray voltage applied was set to 2.0 kV in positive-ion mode. The m/z scan range was 350 to 1,800 for full scan, and intact peptides were detected in the Orbitrap at a resolution of 70,000. Peptides were then selected for MS/MS using NCE setting as 28, and the fragments were detected in the Orbitrap with 17,500 resolution. A data-dependent procedure that alternated between one MS scan followed by 20 MS/MS scans, with 15.0-s dynamic exclusion. Automatic gain control was set at 5E4. Fixed first mass was set as 100 m/z.

### Data Analysis

All raw files were processed using Maxquant search engine (v.1.5.2.8). Tandem mass spectra were searched against mouse database concatenated with reverse decoy database. Trypsin/P was specified as cleavage enzyme allowing up to two missing cleavages. The mass tolerance for precursor ions was set to 20 ppm in the first search and 5 ppm in the main search, and the mass tolerance for fragment ions was set to 0.02 Da. Carbamidomethyl on Cys was specified as fixed modification, and oxidation on Met was specified as variable modifications. The false discovery rate was adjusted to < 1% for all peptide-spectrum matches, and the minimum score for peptides was set to > 40.

### Western Blotting

To validate the aforementioned data, equal amounts of brain and lung protein samples (20 μg) obtained from two mice per group were subjected to gel electrophoresis and transferred to 0.45-μm PVDF membrane (Millipore, United States). The membranes were blocked with 3% non-fat dry milk in TBST (10 mM Tris-HCl, 150 mM NaCl, 0.1% Tween 20, pH 7.6) for 2 h at room temperature. The membranes were incubated with primary antibodies overnight at 4°C. After washing with TBST, the membranes were incubated with the corresponding HRP conjugated secondary antibodies for 2 h at room temperature. After washing with TBST, the membranes were developed with enhanced chemiluminescence reagent. The intensities of bands in the Western blots were quantified by densitometry analysis using Image J software (NIH).

### Immunohistochemical Analysis

At 7 dpi, control (*n* = 2) and infected mice (*n* = 2) were euthanized. The brain and lung samples were obtained and fixed in 10% paraformaldehyde for 48 h. After fixation, paraffin-embedded organs and tissues were cut into 5-μm sections and stained with hematoxylin and eosin (H&E). The expression of selected proteins in the organs was detected in accordance with a standard immunoperoxidase procedure as described previously (Dang et al., 2017).

### Antibodies

The following primary antibodies were used in this study: STAT1 (Cell Signaling Technology Inc., #14994), β-actin (Abcam, Inc., #ab115777), TRIM25 (#abs143488), Cathepsin S (#abs137723), NR3C1 (#abs100327), CDKN1B (#abs100463), Arginase 1 (#abs127734), ISG15 (#abs113675), and NOX2 (cat. #abs124860), which were from Absin Bioscience.

### Bioinformatics Analysis

Gene Ontology (GO) annotation proteome was derived from the UniProt-GOA database^1^. The proteins were classified by GO annotation into three categories: biological process, cellular compartment, and molecular function. For each category, a two-tailed Fisher’s exact test was employed to test the enrichment of the differentially expressed protein against all identified proteins. GO with a corrected *p*-value < 0.05 is considered significant. The Kyoto Encyclopedia of Genes and Genomes (KEGG) database was used to annotate the protein pathways by a two-tailed Fisher’s exact test to test the enrichment of the differentially expressed protein against all identified proteins. The pathway with a corrected *p*-value < 0.05 was considered significant. These pathways were classified into hierarchical categories according to the KEGG website. In this study, we used the WoLF PSORT program to predict the subcellular localization of identified proteins. All differentially expressed protein database accessions or sequences were searched against the STRING database^2^, version 10.5, for protein–protein interactions. STRING defines a metric called “confidence score” to define interaction confidence; we fetched all interactions that had a confidence score > 0.7 (high confidence). The interaction network form STRING was visualized in R package “networkD3”.

### Statistical Analyses

An unpaired *t*-test, as implemented in the limma package in R software (V3.38.3), was performed for the analysis of differences in expression. A *p*-value less than 0.05 was considered statistically significant in this study. Then, the differentially expressed proteins were defined as those with *p*-value < 0.05 and a fold change of EV71/control > 1.2 (significantly upregulated) or < 1/1.2 (significantly downregulated). The volcano plot was used to illustrate changes in protein expressions between control and infected lung tissues, showing log2 fold change of EV71/control (*X*-axis) with log_10_ *p*-value (*Y*-axis). Two-dimensional scatter plot of principal component analysis (PCA) distribution of all samples using quantified proteins was performed by R software (V3.38.3). The heat map of differentially expressed proteins in the brain and lung was illustrated by GraphPad Prism (v. 8.3.0.538). The detailed statistics for each analysis was described in the corresponding sections.

## Results

### Comprehensive Proteomics Characterization of EV71-Infected Brain and Lung Tissues

**FIGURE 1:**
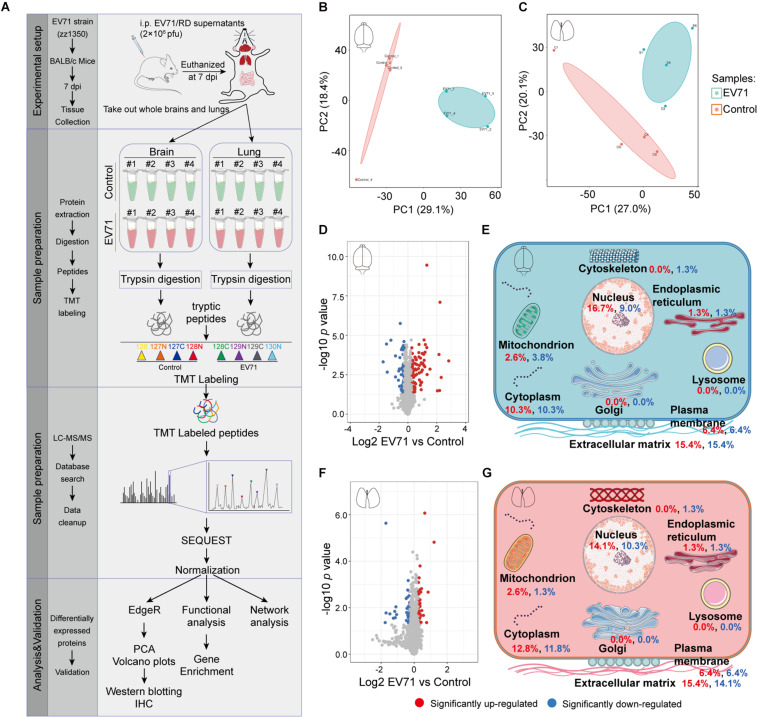
The quantitative proteomic profile of EV71-infected brain and lung tissues. **(A)** Study design of quantitative proteomic analysis for EV71-infected brain and lung tissues. **(B,C)** Principal component analysis of the proteome profile. Biological replicates were generated for each sample, represented by different color points in the figure. **(D,F)** Volcano plots of the −log10 *p* value *vs*. the log2 protein abundance comparisons between brains or lungs from controls and those infected with EV71. Proteins outside the significance threshold lines (*p* value < 0.05 and EV71/control > 1.2 or < 1/1.2) were colored in red (upregulated) or blue (downregulated). **(E,G)** Schematic of changes in the cell components of EV71-infected brain and lung tissues. Red and blue fonts represent up- and downregulated proteins detected in brain or lung tissues from EV71-infected mice compared to controls.

Our previous studies have shown that EV71 infection could cause brain and lung damage (Dang et al., 2017; Jin et al., 2017, 2018a). To investigate the pathological features of this damage, we dissected whole brain and lung tissues from control (*n* = 4) and EV71-infected mice (*n* = 4) at 7 dpi and then performed a proteomic analysis using quantitative mass spectrometry (Figure 1A). A total of 6,410 (with quantitative information from 5,520 proteins) and 6,521 proteins (with quantitative information from 5,497 proteins) were identified in brain and lung specimens from control and EV71-infected mice (Supplementary Tables 1, 2), respectively. The PCA analysis showed that the proteins identified in control and EV71-infected mice formed independent clusters (Figures 1B,C). A total of 148 and 78 proteins were identified to be differentially expressed (*p* < 0.05 and EV71/control > 1.2 or < 1/1.2) in EV71-infected brains (Supplementary Table 3) and lungs (Supplementary Table 4) as compared with the control, respectively. Among these differentially expressed proteins, 89 proteins were upregulated (>1.2-fold) and 59 proteins were downregulated (<1/1.2-fold) in the brain tissues, and 41 proteins were upregulated (>1.2-fold) and 37 proteins were downregulated (<1/1.2-fold) in the lung tissues in response to EV71 infection. Protein expression patterns were visualized using volcano plots (Figures 1D,F). An analysis of subcellular location showed significant changes in proteins that were associated with six different cellular organelles or structures (Figures 1E,G). Most of the differentially expressed proteins were localized in the cell nucleus and cytoplasm, which suggested that these proteins may be related to virus infection-induced gene transcription. Interestingly, among different subcellular localizations, the highest percentage of proteins were associated with the extracellular matrix, suggesting that EV71 infection might result in dysregulation of the extracellular microenvironment in the brain and lung. Taken together, these results suggested that the expression of a wide range of proteins was changed significantly both in the brain and lung after EV71 infection.

### GO Enrichment Analysis Reveals Immune System Response and Platelet Activation

**FIGURE 2:**
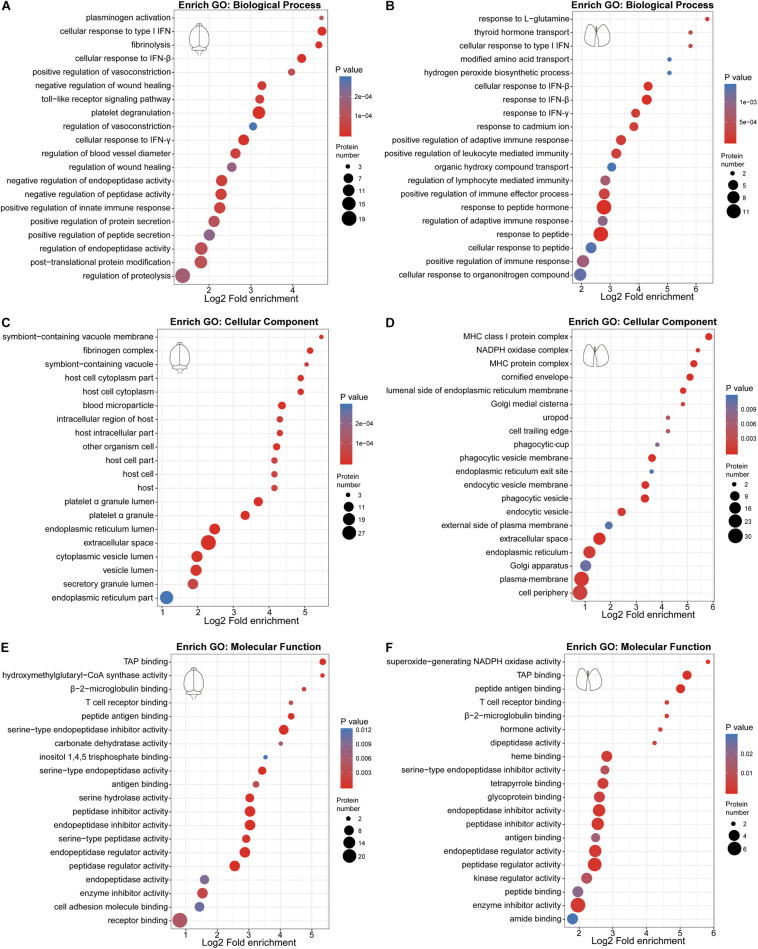
Gene Ontology (GO) enrichment bubble plot of differentially expressed proteins in the three categories. **(A,C,E)** GO enrichment analysis of differentially expressed proteins in the brain. **(B,D,F)** GO enrichment analysis of differentially expressed proteins in the lung.

**FIGURE 3:**
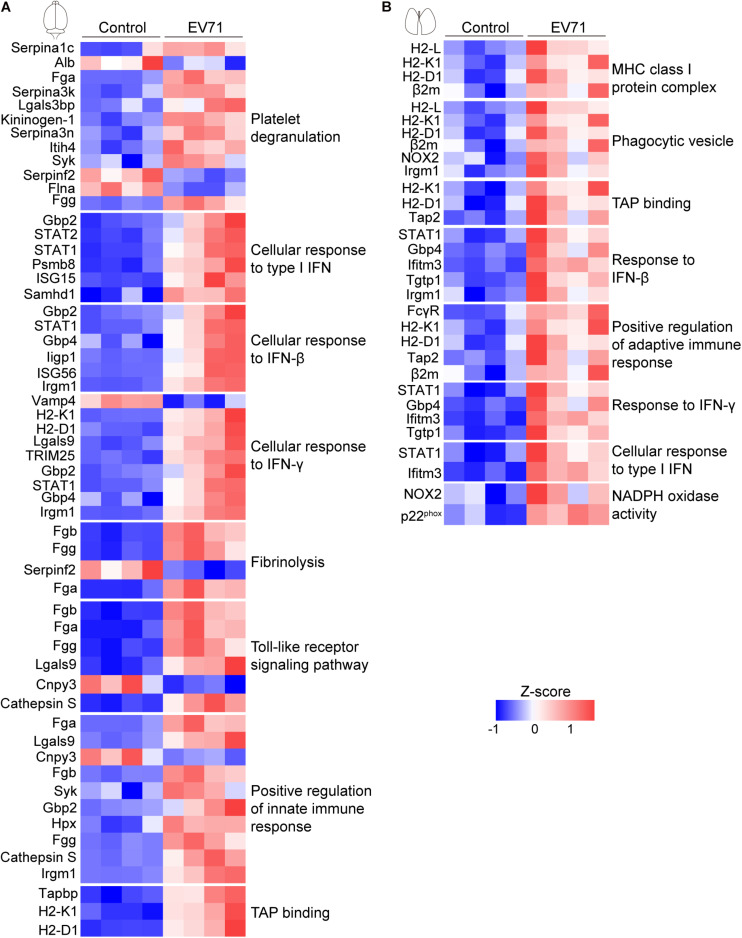
Visualization of changing proteins and their biological pathways. **(A)** Heat map of differentially expressed proteins in the brain (control: *n* = 4, EV71: *n* = 4) that are involved with key biological pathways. **(B)** Heat map of differentially expressed proteins in the lung (control: *n* = 4, EV71: *n* = 4) that are involved with key biological pathways.

We performed GO enrichment analysis of these differentially expressed proteins to compare the host response in the brain and lung. Our results showed that protein alterations in these tissues differed in several main categories (Figure 2). Both the innate immune response, represented by cellular response to type I interferon (IFN), cellular response to IFN-β, response to IFN-β, positive regulation of innate immune response and Toll-like receptor signaling pathway, and the adaptive immune response, represented by cellular response to IFN-γ, response to IFN-γ, regulation of adaptive immune response, regulation of lymphocyte mediated immunity, positive regulation of adaptive immune response, major histocompatibility complex (MHC) class I protein complex, and TAP binding, were activated in the brain and lung sites. As shown in Figures 3A,B, these immune processes contained transcription factors (e.g., *STAT1* and *STAT2*), regulators of innate signaling pathway (e.g., *TRIM25*, *Fgb*, *Fga*, *Fgg*, *Lgals9*, *Cnpy3*, *Gbp2*, *Gbp4*, *Hpx*, and *Syk*), antigen presentation and processing (e.g., *H2-L*, *H2-K1*, *H2-D1*, *Cathepsin S*, β*2m*, *Tap2*, *FcγR*, and *Tgtp1*), and many IFN-stimulated genes (ISGs; e.g., *ISG15*, *ISG56*, *Irgp1*, *Irgm1*, *Psmb8*, and *Ifitm3*). On the one hand, the activation of immune response, both in the brain and lung, could provide the mice with effective immune protection against EV71 infection; on the other hand, it might induce excessive cytokine production, which is consistent with “cytokine storm” in patients at severe and critical stages of HFMD (Duan et al., 2014; Teo et al., 2018).

There were also many differences in the differentially expressed proteins at these two sites. The plasminogen activation, platelet degranulation, fibrinolysis, and fibrinogen complex were only detected in EV71-infected brain tissues (Figures 2A,C), which contained *Fgb*, *Fga*, *Fgg*, *Serpina 1c*, *Alb*, *Serpina 3k*, *Lgals3bp*, *Kininogen-1*, *Serpina 3n*, *Itih4*, *Syk*, *Serpinf2*, and *Flna* (Figure 3A). The NADPH oxidase activity (e.g., *NOX2* and *p22*^*phox*^) was significantly elevated in EV71-infected lungs (Figures 2D,F, 3B), which suggested that an imbalance of oxidation–reduction due to oxidative stress might be involved in lung damage.

### KEGG Enrichment Analysis Reveals Complement and Coagulation Dysfunction, Immune Activation, an Imbalance of Nitrogen Metabolism, and Hypoxia

**FIGURE 4:**
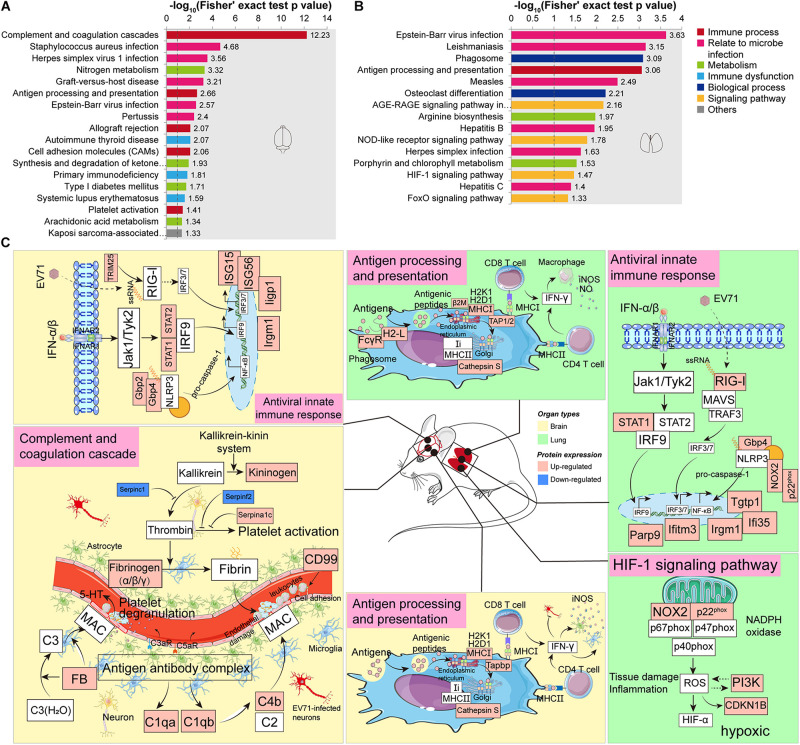
Kyoto Encyclopedia of Genes and Genomes (KEGG) enrichment analysis of significantly dysregulated proteins. **(A)** KEGG enrichment analysis of significantly dysregulated proteins in the brain. **(B)** KEGG enrichment analysis of significantly dysregulated proteins in the lung. **(C)** A hypothetical systems view of the two organs’ responses to EV71 infection. In the brain or lung, the virus and its released RNA could induce an antiviral innate immune response. APCs bind antigens and internalize, process, and express them on their surface for recognition by certain lymphocytes such as CD4 T cells and CD8 T cells. In the brain, the virus infection induces complement activation which contributes to disease by attracting and activating inflammatory cells and by direct damage to endothelial cells through MAC. The endothelial dysfunction leads to the activation of coagulation cascade and platelet through KKS cascade. In the lung, the NADPH oxidase activity was significantly increased in response to EV71 infection, which might activate the HIF-1 signaling pathway, leading to a hypoxic condition. Red boxes, upregulated proteins/pathways; blue boxes, downregulated proteins/pathways.

KEGG enrichment analysis was carried out to determine the principal functions of significantly dysregulated proteins. In the brain, significantly altered proteins by EV71 infection participated primarily in complement and coagulation cascades (*Serpina1c*, *C1qb*, *Fga*, *C1qa*, *Kng1*, *Fgb*, *Serpinf2*, *FB*, *Serpinc1*, *C4b*, and *Fgg*), infectious diseases (*C1qb*, *FB*, *Fgg*, *C1qa*, *C4b*, *Tapbp*, *H2-K1*, *H2-D1*, *Srpk1*, *Srsf5*, *Syk*, *STAT1*, *STAT2*, and *ISG15*), antigen processing and presentation (*Tapbp*, *H2-K1*, *H2-D1*, and *Cathepsin S*), nitrogen metabolism (*Ca5b*, *Ca4*, and *Glul*), cell adhesion molecules (*Ptprc*, *H2-K1*, *H2-D1*, *Cldn3*, and *CD99*), and platelet activation (*Mylk2*, *Fgb*, *Syk*, *Fga*, and *Fgg*) (Figures 4A,C and Supplementary Table 5). The complement (C) system constitutes an important barrier to infection of the human body but can also, when inappropriately activated, cause tissue damage (Stoermer and Morrison, 2011). The complement system is activated *via* multiple pathways, leading to the production of C3a, C5a, and C5b-C9 membrane attack complex (MAC) that are increasingly recognized as mediators of protection or pathology in a variety of viral infections (Stoermer and Morrison, 2011). Previous studies have provided the evidence that the JAK-STAT signaling pathway (*STAT1*, *STAT2*, and *ISG15*), NOD-like receptor (NLR) signaling pathway (*Gbp2* and *Gbp4*), and RIG-I signaling pathway (*TRIM25*) are activated during EV71 infection (Jin et al., 2018b). These innate signaling pathways, in cooperation with ISG (*ISG15*, *ISG56*, *Iigp1*, and *Irgm1*) production, might provide antiviral effects against EV71 infection. Additionally, the JAK-STAT and NLR signaling pathways also mediate pro-inflammatory cytokine production (Jin et al., 2018b). Antigen processing and presentation refer to the processes that occur within a cell that result in fragmentation (proteolysis) of proteins, association of the fragments with MHC molecules, and expression of the peptide–MHC molecules at the cell surface (Cresswell, 2005). The result of these actions is the induction of a T cell response (Cresswell, 2005) and leukocyte adhesion to the site of infection (Pober et al., 2001), causing EV71-related brain damage. T cell-secreted IFN-γ could induce glia cells to produce inducible nitric oxide synthase (iNOS) (Mir et al., 2008). Nitrogen metabolism is related to the synthesis of iNOS, and it has been demonstrated that EV71 pathogenesis may involve iNOS and nitric oxide (NO) (Kao et al., 2004; Dang et al., 2017). As mentioned earlier, EV71-induced BBB disruption may be associated with coagulation dysfunction. Increased coagulation clots (*Fgb*, *Fga*, and *Fgg*) and platelet activation contribute to the formation of thrombin that may interact to increase brain endothelial permeability (Avril et al., 2019).

In the lung, EV71 infection led to changes in the proteins involved in infectious diseases (*H2-L*, *STAT1*, *PI3K*, *Tap2*, *CDKN1B*, *RIG-I*, β*2m*, *FcγR*, *NOX2*, and *p22phox*), phagosome (*H2-L*, *FcγR*, *NOX2*, *p22phox*, *Tap2*, and *Cathepsin S*), antigen processing and presentation (*H2-L*, β*2m*, *Tap2*, and *Cathepsin S*), AGE-RAGE signaling pathway (*STAT1*, *PI3K*, *NOX2*, and *CDKN1B*), arginine biosynthesis (*Arginase-1* and *Otc*), NLR signaling pathway (*STAT1*, *NOX2*, *p22phox*, and *Gbp4*), and HIF-1 signaling pathway (*NOX2*, *PI3K*, and *CDKN1B*) (Figures 4B,C and Supplementary Table 6). Similar to the brain, the JAK-STAT signaling pathway (*STAT1*), RIG-I signaling pathway (*RIG-I*), and NLR signaling pathway (*Gbp4*, *NOX2*, and *p22phox*) could also provide antiviral effects against EV71 infection through inducing ISGs (*Parp9*, *Ifi35*, *Iigm1*, *Ifitm3*, and *Tgtp1*). In addition, significantly upregulated proteins enriched in antigen processing and presentation suggested a T cell response. IFN-γ derived from T cells provides antiviral effects against virus infection as well as stimulates macrophages to produce iNOS and NO that possess pro-inflammatory effects (Sareila et al., 2006). Arginase-1 is an important component of nitrogen metabolism, regulating arginine availability during immune responses and nitric oxide synthase activity (Durante et al., 2007; Vanhoutte, 2008). Arginase-1 deficiency can facilitate inflammatory reaction and NO production (Wijnands et al., 2014). Arginase-1 biosynthesis was significantly reduced in response to EV71 infection in the lung site. The above-mentioned data indicated the presence of inflammation in the lung. It is well known that innate immunity depends on rapid recognition to eliminate invading pathogens. This entails sequestration of pathogenic invaders into phagosomes that promptly acquire microbicidal and degradative properties (Pauwels et al., 2017). The overlap of the dysregulated proteins involved in phagosomes and antigen processing and presentation suggested that the phagocytic cells might also act as antigen-presenting cells (APCs).

### Validation of Proteomic Approach by Immunohistochemical Staining and Western Blotting

**FIGURE 5:**
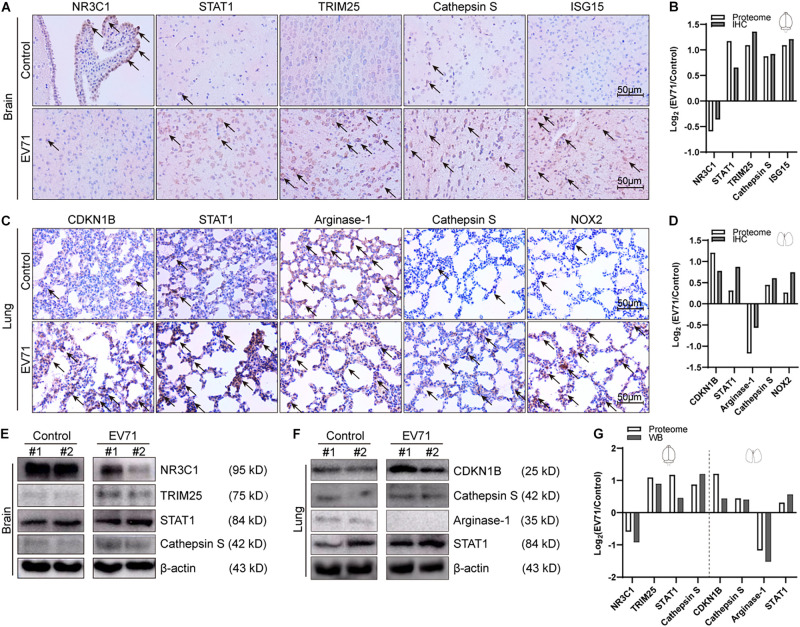
Comparison of laboratory measurements and proteome. At 7 dpi, EV71-infected mice (*n* = 4) and controls (*n* = 4) were euthanized, and brain and lung tissues were taken out for immunohistochemical (IHC) staining (*n* = 2) and Western blotting (*n* = 2). **(A)** Immunohistochemical analyses of NR3C1, STAT1, TRIM25, Cathepsin S, and ISG15 in EV71-infected brain tissues and control brain tissues. Black arrows represent positively stained areas (brown). **(B)** Comparison of intensities of IHC staining and proteomic data (TMT reporter ion intensities) in the brain. **(C)** Immunohistochemical analyses of CDKN1B, STAT1, Arginase-1, Cathepsin S, and NOX2 in EV71-infected lung tissues and control lung tissues. **(D)** Comparison of intensities of IHC staining and proteomic data (TMT reporter ion intensities) in the lung. **(E)** Western blotting analyses of NR3C1, TRIM25, STAT1, and Cathepsin S in EV71-infected brain tissues and control brain tissues. **(F)** Western blotting analyses of CDKN1B, Cathepsin S, Arginase-1, and STAT1 in EV71-infected lung tissues and control lung tissues. **(G)** Comparison of intensities of immunoblots and proteomic data (TMT reporter ion intensities) in the brain and lung. All laboratory measurements were repeated at least three times. Data are displayed as log2 (EV71/control).

To validate our proteomic approach, we compared the expression levels of dysregulated proteins discovered by proteomics (TMT reporter ion intensities) by immunohistochemical staining (Figures 5A–D) or Western blotting (Figures 5E–G). The dysregulated proteins selected for validation were mainly based on the GO and KEGG enrichment pathways. As shown in Figures 5A,B,E,G, the changes of expression of NR3C1, STAT1, TRIM25, Cathepsin S, and ISG15 in the brain were consistent with the data from the proteomic analysis. The alterations of expression of CDKN1B, STAT1, Arginase-1, Cathepsin S, and NOX2 in the lung were also consistent. Therefore, the results by Western blotting or IHC analysis confirmed changes of protein expression identified by TMT quantification.

### Protein–Protein Interaction Network Analysis Reveals a Greater Interference of Signal Molecules in the Brain

**FIGURE 6:**
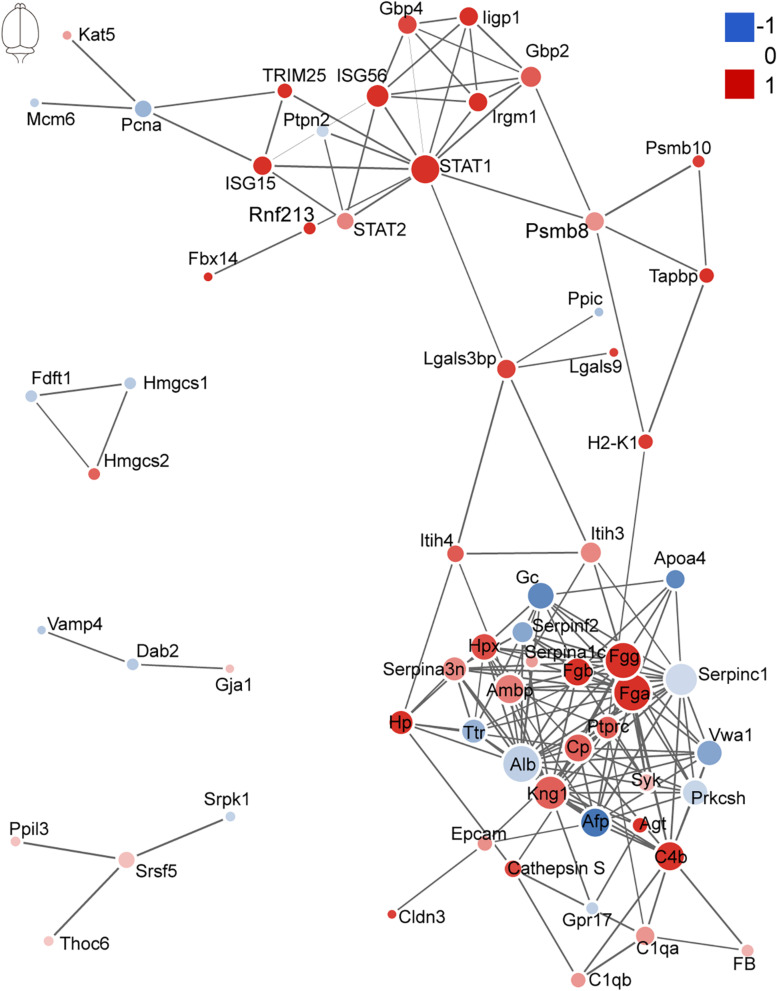
Protein–protein interaction analysis of dysregulated proteins in the brain. The size of the nodes corresponds to the number of interacting proteins, whereas the different colors represent the regulation type and magnitude of protein changes. Red color represents upregulation, and blue color represents downregulation. The full list of key driver proteins can be found in Supplementary Table 3.

**FIGURE 7:**
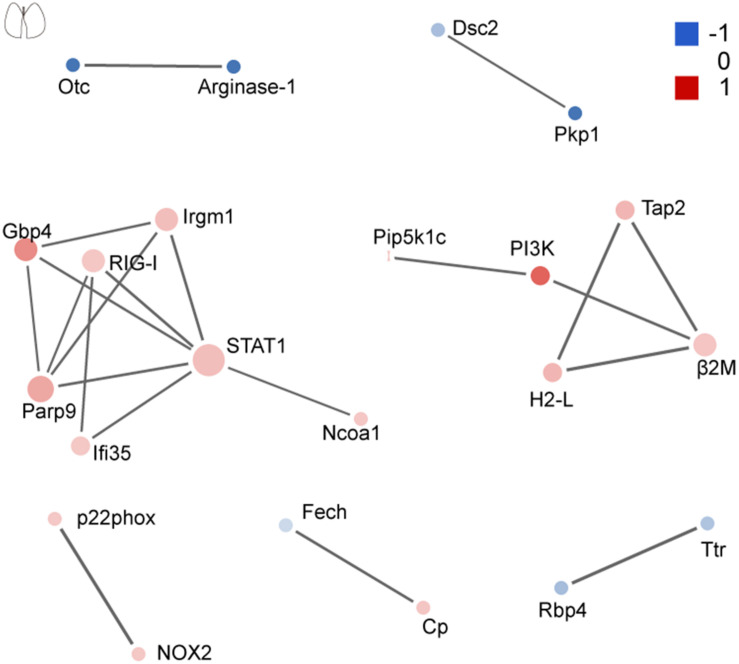
Protein–protein interaction analysis of dysregulated proteins in the lung. The size of the nodes corresponds to the number of interacting proteins, whereas the different colors represent the regulation type and magnitude of protein changes. Red color represents upregulation, and blue color represents downregulation. The full list of key driver proteins can be found in Supplementary Table 4.

The network of PPI helps to understand the potential molecular mechanisms of brain and lung damage induced by EV71 infection. In this study, a PPI network was constructed with STRING. In Figure 6, among the 74 differential candidates from EV71-infected brain, *STAT1*, coagulation clots (*Fga* and *Fgg*), *Alb*, *Kininogen-1*, *C4b*, and *Afp* were identified as key driver proteins. In Figure 7, among 22 differential candidates in the lung, STAT1 was the only key driver protein. These data suggested that EV71 infection caused a greater interference of signal molecules in the brain, which is linked to the severe CNS damage in HFMD cases. Despite the elimination of polio in most countries, EV71 has been recognized as an emerging neurotropic virus, which leads to neurological complications with severe CNS damage (Huang et al., 1999; Solomon et al., 2010; Chua and Kasri, 2011; Xing et al., 2014; Yang et al., 2017). In human infections, CNS complications and its related pulmonary edema are considered as the main cause of death (Solomon et al., 2010; Xing et al., 2014; Yang et al., 2017). Notably, STAT1 was the key driver protein both in the brain and lung. It has been reported that STAT1 deficiency can enhance the susceptibility of scavenger receptor class B, member 2 (SCARB2) transgenic mice to EV71 (Liou et al., 2016), indicating that STAT1 plays an important role in EV71 pathogenesis. Our data together showed that dysregulated proteins interacted with each other and contributed to EV71-associated brain and lung damage.

## Discussion

In the past decades, studies on epidemiology and pathology provided descriptive information in terms of the clinical pathology of EV71-associated encephalitis and respiratory disorders (Lum et al., 1998; Solomon et al., 2010; Zhang et al., 2012; Tu et al., 2015). Moreover, various animal models were also used to explore the possible mechanisms of EV71 pathogenesis (Liou et al., 2016; Jin et al., 2018a). However, the landscape of EV71-induced molecular pathogenesis remains to be elucidated. CNS is the main target of EV71, and CNS damage can be found in patients with severe symptoms of EV71 infection (Ho et al., 1999; Huang et al., 1999; Solomon et al., 2010; Yang et al., 2017). Our results showed that more signaling proteins were affected in the brain rather than in the lung site. As known to all, both innate and adaptive immunity play a central role in our host defense against pathogens. We found that both the innate and adaptive immune systems were activated in the brain and lung from EV71-infected mice. The activated immune system provides protection against virus infection; however, it may also cause a “cytokine storm,” leading to increased inflammation and tissue damage (Duan et al., 2014; Teo et al., 2018). Notably, activation of the JAK-STAT, NLR, and RIG-I signaling pathways was observed both in the brain and lung. These critical signaling pathways provide potential targets for drug design. In addition, as EV71-infected brains and lungs shared the key driver protein, STAT1, more attention should be paid to STAT1, an important regulator during innate and adaptive immune responses.

Another important finding was the complement and coagulation dysfunction in EV71-infected brains. The brain is known as an immune-privileged organ that is uniquely placed in the body. Evolving knowledge suggests that the BBB and the complement system are two systems involved in brain defense and homeostasis (Alexander, 2018). The complement system is extremely important in the brain that is an immune-privileged section with restricted adaptive immunity, acting as a local surveillance system protecting the brain from virus infection and participates in tissue homeostasis and repair (Ricklin et al., 2016). The BBB, on the other hand, serves as a local barrier protecting the brain from circulating infected cells and toxins (Abbott et al., 2010). Although complement components are mainly generated in the liver and then transported into circulation, established evidence suggests that the constituent cells of the BBB, such as endothelial cells and astrocytes as well as neurons, can synthesize complement components (Rus and Niculescu, 2001). Complement activation products, C3a and C5a, cause the activation of constituent cells of the BBB and increase the infiltration of inflammatory cells into the brain, resulting in a second tissue damage and organ dysfunction (Alexander, 2018; Lee et al., 2019; Dalakas et al., 2020). The BBB disruption that can occur with complement dysregulation or be exacerbated by complement hyper-activation exposes the brain to circulating toxins. Similarly, inhibition of the complement activation can alleviate the pathology, leading to an improved outcome (Flierl et al., 2009; Alexander, 2018). As mentioned earlier, endothelial dysfunction induced by EV71 infection provides a possible mechanism for coagulation disorders in the brain (Wang et al., 2012b). Neonatal sepsis is frequently reported in HFMD patients (Ooi et al., 2010), and sepsis can induce excessive inflammatory cytokines, which, in turn, activate the coagulation cascades (Simmons and Pittet, 2015). Hypercoagulation can result in microvascular thrombosis as well as oxygen deficiency, leading to tissue damage (Leng et al., 2020). Complement and coagulation dysfunction was never reported in HFMD patients; thus, this finding provided a unique insight into the pathogenesis of EV71. Complement and coagulation pathways may pose potential targets for drug design to treat EV71 infection.

In addition to the proteins mentioned above, some key differentially expressed proteins may also contribute to EV71 pathogenesis. The degranulation of platelets releases pathogenic serotonin (5-HT) and pro-inflammatory mediators (Blockmans et al., 1995), leading to damage of the BBB. It has been reported that EV71 can directly infect endothelial cells (Wang et al., 2012b), suggesting that platelet activation within the brain site in the absence of trauma may be initiated by vascular injury (Broos et al., 2011). In addition, immune complexes can also induce platelet activation (Cloutier et al., 2018). Moreover, platelet activation has been linked to vascular leak induced by virus infection (Malavige and Ogg, 2017; Hottz et al., 2020). Plasminogen activation, fibrinolysis, and fibrinogen complex associate with the formation of thrombin, which has been reported in viral pathogenesis (Leng et al., 2020). Thrombin and fibrinogen are epidemiologically and mechanistically linked with diseases with an inflammatory component (Davalos and Akassoglou, 2012). It has been reported that antibody-dependent enhancement during EV71 infection is mediated in part by Fcγ receptors (FcγRs) in immune cells, suggesting that the blockage of FcγR in antigen-presenting cells may provide a strategy for the treatment of EV71 infections (Wang et al., 2010). The hypoxia-inducible factor 1 (HIF-1) signaling pathway plays an essential role in the maintenance of oxygen homeostasis (Semenza, 2007). In this study, we found that the HIF-1 signaling pathway was activated in the lung site, which may lead to a hypoxic condition. Previous studies suggest that the HIF-1 signaling pathway contributes to acute lung injury by viral infections (Mermis et al., 2011; Zhao et al., 2020). A hypoxic condition may associate with respiratory failure that was reported in critical HFMD patients and deaths with pulmonary edema (Chan et al., 2000; Kao et al., 2004). The renin–angiotensin system (RAS) plays a vital role in multiple infectious diseases through augmenting inflammatory reactions (Gao et al., 2020). Angiotensinogen (Agt), the substrate of the renin–angiotensin cascade, was significantly increased in EV71-infected brains. It is possible that RAS might participate in neuroinflammation by EV71. Nuclear receptor subfamily 3, group C, member 1 (NR3C1) is known as a glucocorticoid receptor (Juszczak and Stankiewicz, 2018), which was significantly downregulated in EV71-infected brains. NR3C1 regulates genes that control the development, metabolism, and immune response (Juszczak and Stankiewicz, 2018), but it is not clear whether the downregulation of NR3C1 is predominantly beneficial or detrimental during brain damage. One of the matrix metalloproteinases (MMPs), MMP1, was identified to be upregulated in EV71-infected brains, indicating that MMPs may contribute to BBB damage during EV71 infection (Leib et al., 2000).

In summary, we characterized the pathogenesis of EV71 at the molecular level using an animal model for the first time. Our results indicated that immune activation, complement and coagulation dysfunction, platelet activation, imbalance of nitrogen metabolism, and hypoxia could be involved in the pathogenesis of EV71, which explain the major clinical manifestation of hyperinflammatory status of severe HFMD cases. These findings provided insights into the pathogenic mechanisms of EV71 infection and further understanding of HFMD-associated encephalitis and respiratory disorders.

## Data Availability Statement

Data that support the findings of this study are available via ProteomeXchange (http://www.ebi.ac.uk/pride) with identifier PXD023819. Source data are provided with this manuscript.

## Ethics Statement

The animal study was reviewed and approved by the Life Science Ethics Review Board of Zhengzhou University.

## Author Contributions

YJ, SC, and GD conceived and designed the research. YJ, DL, TS, YD, YG, RD, and WJ directly participated in the experiment. YJ drafted the manuscript. YJ, SC, GD, WZ, and HY participated in the design of the study and modification of English grammar. All authors read and approved the final manuscript.

## Conflict of Interest

The authors declare that the research was conducted in the absence of any commercial or financial relationships that could be construed as a potential conflict of interest.

## Footnotes

1 http://www.ebi.ac.uk/GOA/

2 https://string-db.org/
